# Recurrent Streptococcus Pneumoniae Meningitis in a Child with Split Hand and Foot Malformation and Undiagnosed Mondini Dysplasia

**DOI:** 10.1007/s10882-015-9460-2

**Published:** 2015-11-12

**Authors:** Mazur-Melewska Katarzyna, Szydłowski Jarosław, Jończyk-Potoczna Katarzyna, Służewski Wojciech, Figlerowicz Magdalena

**Affiliations:** Department of Infectious Diseases and Child Neurology, Karol Marcinkowski University of Medical Sciences in Poznań, Szpitalna Street 27/33, 60-578 Poznań, Poland; Pediatric ENT Department, Karol Marcinkowski Poznan University of Medical Sciences, Poznań, Szpitalna Street 27/33, 60-578 Poznań, Poland; Pediatric Radiology Department Chair of Radiology, Karol Marcinkowski University of Medical Sciences, Poznań, Szpitalna Street 27/33, 60-578 Poznań, Poland

**Keywords:** Mondini dysplasia, Recurrent meningitis, Split hand and foot malformation

## Abstract

Recurrent bacterial meningitis is a life-threatening infection of the central nervous system that is mostly connected with anatomical abnormalities of the skull, chronic parameningeal infections and immunodeficiencies. It’s rarely seen, but when it occurs an extensive investigation should be carried out to discover the responsible factor, so that further episodes can be prevented. We report on a child with split hand and foot (SHFM), confirmed incorrect karyotype 46, XY, t(7:12)(q21.2;q21.3) and a fourth episode of fulminant meningitis caused by penicillin-resistant *Streptococcus pneumoniae*. After a broad evaluation of factors predisposing to recurrent meningitis, the undiagnosed malformation of his inner and middle ears - Mondini dysplasia was found. We suggest examining all children with SHFM for hearing impairment before they develop recurrent meningitis. The time when the radiological procedure for searching for inner ear dysplasia should be performed could be a controversial issue: before or after the first episode of meningitis. From the epidemiological point of view, high-resolution computer tomography scanning of the temporal bones should be considered after the diagnosis of deafness in a child with SHFM related to 7q21 deletion.

## Introduction

Bacterial meningitis is a severe, life-threatening infection of the central nervous system (CNS) that is associated with high rates of significant disability and morbidity in children. Recurrent bacterial meningitis is defined as two or more episodes of meningitis caused by a different bacterial organism or, alternatively, a second or further episode caused by the same organism with a greater-than-3-week interval after the completion of therapy (Tebtuegge and Curtis 2008). It’s rarely seen, but when it occurs an extensive investigation should be carried out to discover the responsible factor, so that further episodes can be prevented (Kendirli et al. 2006). The predisposing factors for recurrent bacterial meningitis can be categorized into congenital and acquired and both groups can be divided into anatomical abnormalities, chronic parameningeal infections and immunodeficiencies such as asplenia, human immunodeficiency virus infection and complement system deficiency (Janocha-Litwin and Simon 2013). Different cranial and spinal anatomic defects can facilitate the migration of bacteria to cerebrospinal fluid (CSF). Another frequent abnormality is congenital inner ear malformations with communication between the subarachnoid space and the air-filled cells or paranasal sinuses, which is the leading cause of recurrent bacterial meningitis (Yang et al. 2008). In patients with congenital defects of different organs and recurrent meningitis, evaluation of inner ear malformation should be the first diagnostic step in the protection against further episodes.

We report on a 13-year-old boy with split hand and foot malformations (SHFM) who suffered from recurrent *Streptococcus pneumoniae* meningitis due to undiagnosed Mondini dysplasia of his ears.

## Case Report

### Participant

A 13-year-old boy with SHFM was admitted to our department with his fourth episode of purulent meningitis. His family history was significant: he was born as the third child in the family; his sister is alive; his brother, who had a congenital heart malformation and Down’s syndrome, died as an infant. His two cousins had Say-Meyer syndrome. His mother had contact with chemical disinfectants during the first two months of her pregnancy. The boy was born in the 38th week of pregnancy with Apgar scale 9 congenital malformation of all four limbs. When he was 1 year old, his family recognized that he had hearing problems but they did not seek any professional help. In 2006, his chromosome analysis confirmed incorrect karyotype 46, XY, t(7:12)(q21.2;q21.3). The boy had reciprocal translocation between chromosome pairs 7 and 12.^***^

The first episode of invasive streptococcal disease was diagnosed in 2008 when the patient was 6 years old. It was a complication of an inflammation of his right ear. After that, the boy developed two further episodes of meningitis: in 2011, without confirmed aetiology, and in 2012, again streptococcal. In 2011, due to his recurrent infections of the central nervous system, the boy had a consultation with a neurosurgeon and underwent an magnetic resonance imaging (MRI) of the brain and lumbar column, but the radiologist did not find any abnormalities of these structures.

### Procedure

**Fig. 1:**
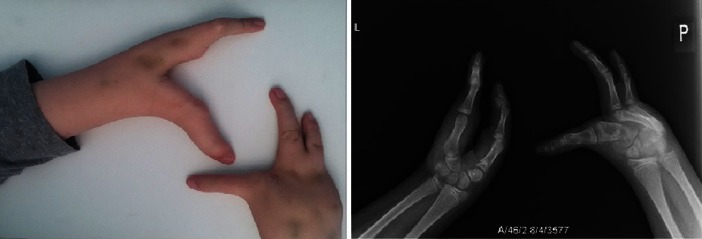
Bilateral cleft wrist and metacarpus with fingers and metacarpal hypoplasia. The remaining 2 metacarpal bones in the right hand and 2 in the left hand. 3 finger bones in the right hand and 2 bones in the left in a “lobster-like” position

**Fig. 2:**
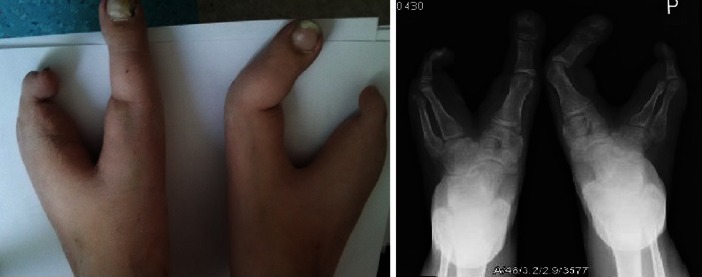
Bilateral cleft tarsus and metatarsus with toes and metatarsal bone hypoplasia. The remaining 3 metatarsal bones and 2 toes in „lobster-like” position. Medial nail phalanges splited

In April 2015, the boy started to complain of pain in his left ear. After three hours the laryngologist confirmed the presence of an inflammation in his left ear and prescribed an oral antibiotic. The pain was so severe that the mother took the child to the hospital two hours later. At the time of admission the general status of the child was good: he presented neither a fever nor pathological neurological symptoms. His leucocyte count was 18.7 G/uL; C-reactive protein– 0.39 mg/dL; procalcitonin – 43.07 ng/mL. The patient received a first dosage of intravenous ceftriaxone. Three hours later the general status of the child had worsened: he started to vomit and complained of a headache. He presented positive meningeal signs. His leucocyte count increased to 27.95 G/ul; C-reactive protein – 12.7 mg/dL. A lumbar puncture was performed and confirmed the diagnosis of purulent meningitis (leu – 137 cells/mm3 with 96 % of neutrophils; protein – 3.77 g/L; glucose – 0.01 mmol/L). Therapy with ceftriaxone and vancomycin was started. The next morning the child was sent to actual department. At the time of admission the boy presented severe hyperaesthesia of the skin, neck stiffness of 7 cm and a positive Kernig’s sign. Physical examination of the body revealed median clefts of both hands and feet (Figs.1 and 2). Other systemic examinations were normal except for a mild systemic murmur in the heart apex. The child presented appropriate-to-age development, but because he was deaf and without speech he used only sign language. The laboratory findings were worse: the leucocyte count was 38.26 G/uL with 98 % of neutrophils; C-reactive protein – 40.20 mg/dL; procalcitonin – 43.89 ng/mL. The blood and CSF culture confirmed penicillin-resistant *Streptococcus pneumoniae* with low sensitivity to vancomycin infection. The patient received high-intensity treatment with cefotaxym (107 mg/kg/day), vancomycin (4 × 10 mg/kg/day) and meropenem (3 × 1 g/day). Over the next few days we observed a slow improvement of the patient’s general status. His therapy was complicated by two episodes of generalized tonic-clonic seizures, which were observed on the 10th and 16th days of treatment. Post-infection epilepsy was diagnosed and levetiracetam was induced.

### Methods

**Fig. 3:**
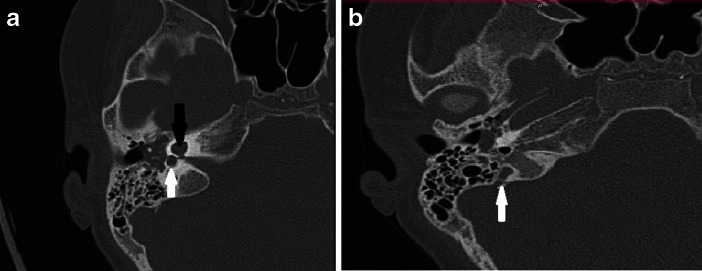
**a.** CT scan, temporal bone, axial view shows preserved basal turn but absence of the apical turns of the cochlea (black arrow), dilated vestibule (white arrow). **b.** CT scan, temporal bone, axial view shows enlarged vestibular aqueduct (white arrow)

**Fig. 4:**
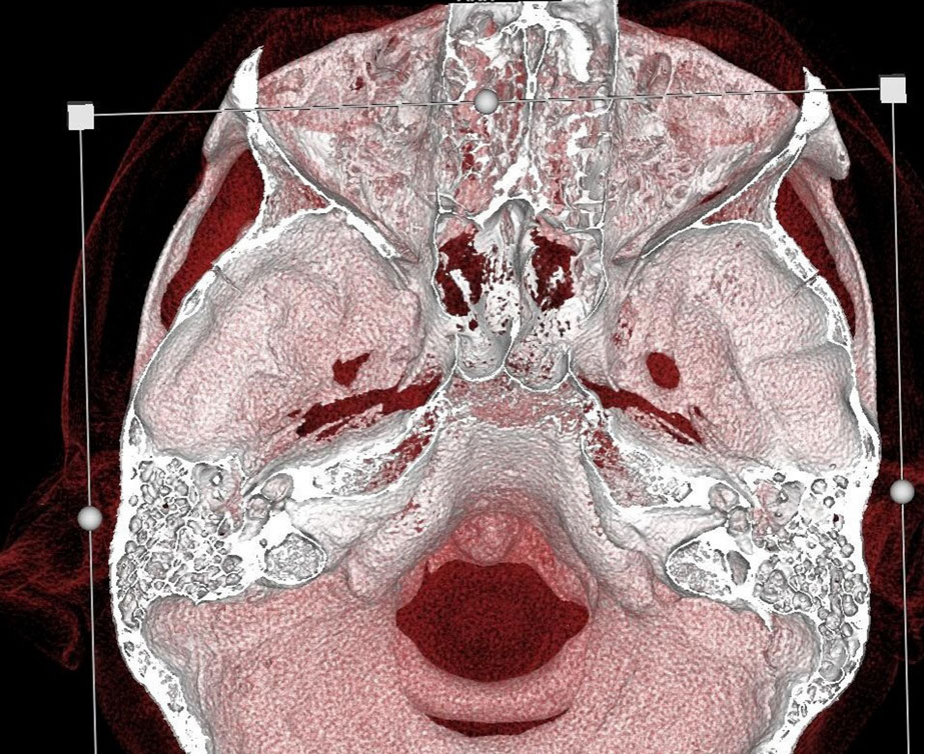
CT- Volume Rendering Techniques scan of temporal bone - Mondini Malformation

**Fig. 5:**
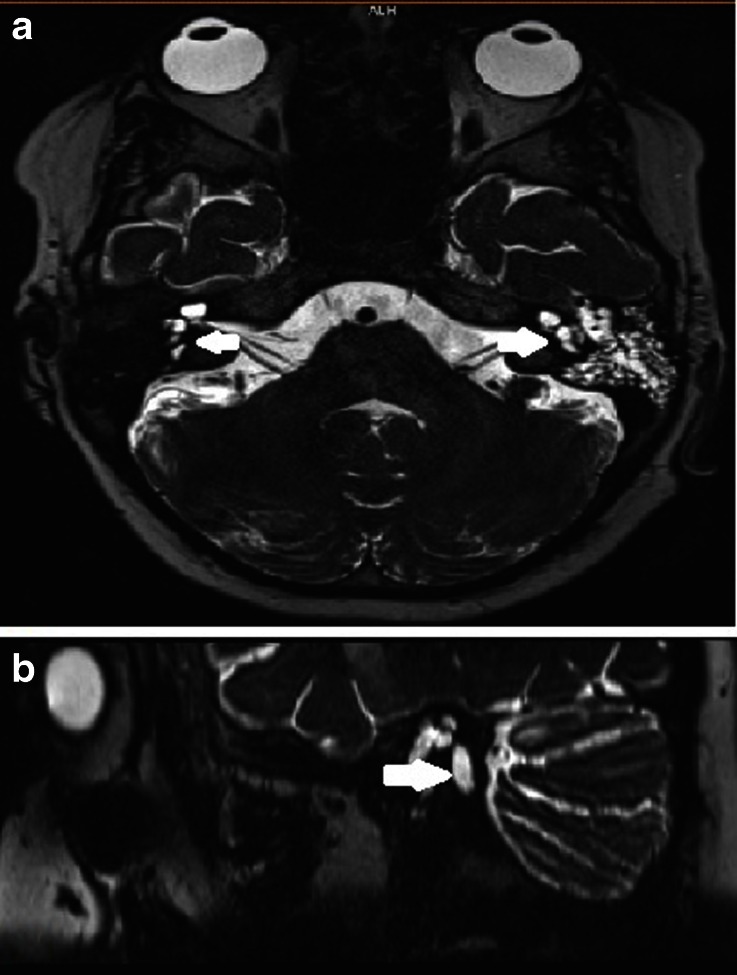
**a.** MRI scan, axial view, T2 weight shows absence of the apical turns and preserved basal turn of the cochlea (black arrow) and enlarged vestibule(white arrow); **b.** MRI scan, sagital view shows enlarged vestibular aqueduct (white arrow)

During his hospitalization we tried to find the reason for his recurrent pneumococcal meningitis. Immunological analysis did not present any abnormalities, and the levels of immunoglobulins G, A and M were correct. The cytometric analysis of the leucocytes presented increased absolute values of lymphocytes CD4-T, CD8-T and B with normal percentages. The expression of adhesive molecules (CD11a, CD11b, CD11c, CD18) was normal. The high-resolution, computer tomography (CT) (axial, coronal, and sagittal planes) of both temporal bones was performed (128-layer Somatom Definition AS apparatus, Siemens, Germany). This indicated: preserved basal turns and absence of the apical turns of the both cochleas, vestibulums wider than usual (Figs. 3 and 4). The clinical suggestion of inner ear abnormality was confirmed – Mondini dysplasia. The malformation was also confirmed in magnetic resonance imagination (3-Tesla MAGNETOM Spectra scanner, Siemens Healthcare, Erlangen/Germany) (Fig. 5).

### Results

Two months after the 4th episode the child had the left subtotal petrosectomy performed to protect him against next recurrent bacterial meningitis. A postauricular S-shaped skin incision was done from the temporal region to the mastoid tip. Mastoid periosteal flap was created and external auditory canal was closed as a “blind sac”. Complete mastoidoepitympanectomy was performed with skeletonisation of the dura, sigmoid sinus and facial nerve. The mastoid cells (retrosigmoid, retrofacial, antral, retrolabyrinthine, supra-labyrinthine, infralabyrinthine, supratubal and pericarotid) were all exenterated. The mucosa of the bony Eustachian tube was removed with the diamond burr and coagulated with bipolar forceps. The Eustachian tube was obliterated with fibrin patch and bone wax. Stapes superstructure was removed. The middle ear cavity was obliterated with abdominal fat harvested from the lower quadrant of the abdomen. Both wounds: abdominal and parietal were closed in layers using 3.0 vicryl subcutaneously and monocryl 3.0 to the skin. The patient’s post-operative course was unremarkable.

## Discussion

Mondini dysplasia and the other forms of congenital inner ear malformations are responsible for a large proportion of recurrent bacterial meningitis. The predisposition to develop bacterial meningitis in these conditions was described by Ozcan et al. as the result from a fistulous connection between the CSF spaces and the middle ear, which in turn is connected to the nasopharynx via the Eustachian tube (Ozcan et al. 2010). Mondini dysplasia is thought to be the result of developmental abnormality around the seventh week of the embryonic development. The cochlea characteristically consists of one and a half turns instead of the normal two and a half turns in association with a defect of the interscalar septum. The modiolus and bone spiral lamina are mostly hypoplastic. The cochlear duct is shortened and dilated and the stria vascularis is often atrophic. The cortical mostly absent and the number of spiral ganglion cells is significantly reduced. The endolymphatic duct and the vestibule and semicircular canals might also be enlarged. CSF leakage can occur primarily through a deficient oval window or stapes footplate in the middle ear (Tebtuegge and Curtis 2008, Ozcan et al. 2010). As was reviewed by Ohlms et al. on 39 patients, Mondini dysplasia is often associated with meningitis caused by *S. pneumoniae* or *Haemophilus influenzae* (Ohlms et al. 1990). Unfortunately only three of the analysed 39 cases were diagnosed after the first episode of central nervous system infections, and the rest of the patients developed between 2 and 20 episodes before the diagnosis of malformation (Jindal et al. 2009).

Mondini dysplasia is mostly seen as a unilateral malformation but there are a few observations of bilateral processes as our patient presented. It can be an isolated malformation but has been described in association with DiGeorge syndrome, Klippel-Feil syndrome and Pendred’s syndrome. It has also been presented as an additional malformation in patients with split hand and foot malformation, which is the large heterogeneous group of congenital limb maldevelopments (Jindal et al. 2009). One of them (SHFM1), associated with translocation of the 7q21 chromosome, is predisposed to coexist with sensorineural deafness in 35 % of cases (Elliott and Evans 2006). In this group of patients, Mondini dysplasia was found (Wieland et al. 2004). For this reason we suggest testing children with SFHM for hearing impairment. The time when the radiological procedure for searching for Mondini dysplasia should be performed could be a controversial issue: before or after the first episode of meningitis. From the epidemiological point of view, high-resolution computer tomography scanning of the temporal bones should be considered after the diagnosis of deafness in a child with SHFM related to 7q21 deletion.
